# 

**Published:** 1865-07

**Authors:** 


### ORIGINAL CONTRIBUTIONS.

ART. XXV. CASE OF PUERPERAL PERITONITIS. By A. NILES, M.D., Quincy, Ill. (Presented to the Illinois State Medical Society.) ........ 385

ART. XXVI. ABNORMAL FŒTUS. By F. R. PAYNE, M.D., Marshall, Ill., ........ 390

### PROCEEDINGS OF SOCIETIES.

American Medical Association. Sixteenth Annual Convention, ........ 391
- Dr. Bigelow's Address, ........ 392
- Dr. Davis' Address, ........ 396
- Papers and Reports from Special Committees ........ 413
- Volunteer Papers, ........ 413
- New Members Elected, ........ 417
- List of Officers and Committees for 1866, ........ 420
- Resolutions on the death of Dr. Knight, Dr. Charles Hooker, and Dr. Benjamin Silliman, ........ 421
- Speech of Dr. Davis at Long Island, ........ 424
- Speech of the Governor of the Commonwealth, ........ 426
- Report of the Committee on the Case of Montrose A. Pallen, ........ 427
- Farewell Address of the President, Dr. Davis, ........ 430

### SELECTIONS.

The Proofs that Lithotrity is an Eminently Successful Operation. By H. Thompson, F.R.C.S., Surgeon Extraordinary to the King of the Belgians, &c., ........ 433

Quackery in Turkey, ........ 432

Small-Pox in Algiers, ........ 432

### EDITORIAL.

Editorial Correspondence.—Letter from Dr. Davis, ........ 437

American Medical Association, ........ 441

Surgeon-General Barnes and Homœopathy, ........ 442

The New Dispensatory, ........ 442

Mortality of Chicago for May, 1865, ........ 443

Electrical Anæsthesia, ........ 444

Surgeons Wanted for the Army at Louisville, Tenn., ........ 445

Health of the Pacific Coast, ........ 445

A Simple Opthalmoscope, ........ 445

Treatment of Hydrocele, ........ 445

Will Oleum Tanaceti Cause Abortion, ........ 464

